# Dichlorido(methanol-κ*O*)[2-(2-pyridyl­meth­oxy)-1,10-phenanthroline-κ^3^ *N*,*N*′,*N*′′]manganese(II)

**DOI:** 10.1107/S1600536808018631

**Published:** 2008-06-25

**Authors:** Hong Liang Li, Hou Chao

**Affiliations:** a Department of Chemistry, Dezhou University, Dezhou Shandong, Dezhou 253023, People’s Republic of China; b Department of Chemistry, Shandong Normal University, Jinan 250014, People’s Republic of China

## Abstract

In the title mononuclear complex, [MnCl_2_(C_18_H_13_N_3_O)(CH_4_O)], the Mn^II^ ion assumes a distorted octa­hedral geometry. There is a π–π stacking inter­action between the phenanthroline ligand and the pyridine ring of a neighboring complex [centroid-to-centroid distance 3.5518 (13) Å]. The crystal structure also contains weak inter­molecular O—H⋯Cl hydrogen bonds that link neighboring complex mol­ecules into a one-dimensional chain along the *b* axis.

## Related literature

For related structures, see: Liu *et al.* (2008 ▶); Li *et al.* (2008 ▶).
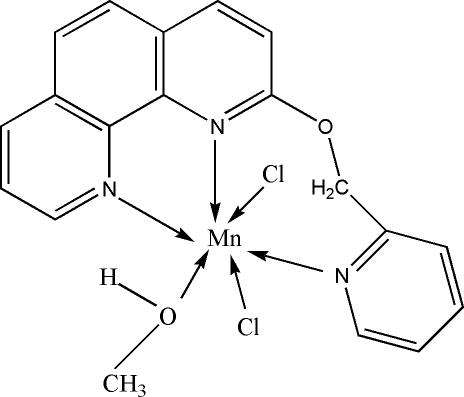


## Experimental

### 

#### Crystal data

[MnCl_2_(C_18_H_13_N_3_O)(CH_4_O)]
*M* *_r_* = 445.20
Monoclinic, 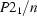

*a* = 10.0390 (16) Å
*b* = 13.667 (2) Å
*c* = 13.583 (2) Å
β = 92.874 (2)°
*V* = 1861.2 (5) Å^3^
*Z* = 4
Mo *K*α radiation
μ = 1.02 mm^−1^
*T* = 298 (2) K
0.38 × 0.18 × 0.13 mm

#### Data collection

Bruker SMART APEX CCD diffractometer
Absorption correction: multi-scan (*SADABS*; Sheldrick, 2008 ▶) *T*_min_ = 0.699, *T*_max_ = 0.879
10717 measured reflections
4048 independent reflections
3278 reflections with *I* > 2σ(*I*)
*R*_int_ = 0.033

#### Refinement

*R*[*F* ^2^ > 2σ(*F* ^2^)] = 0.036
*wR*(*F* ^2^) = 0.090
*S* = 1.01
4048 reflections
245 parameters
1 restraint
H-atom parameters constrained
Δρ_max_ = 0.28 e Å^−3^
Δρ_min_ = −0.26 e Å^−3^

### 

Data collection: *SMART* (Bruker, 1997 ▶); cell refinement: *SAINT* (Bruker, 1997 ▶); data reduction: *SAINT*; program(s) used to solve structure: *SHELXTL* (Sheldrick, 2008 ▶); program(s) used to refine structure: *SHELXTL*; molecular graphics: *SHELXTL*; software used to prepare material for publication: *SHELXTL* and local programs.

## Supplementary Material

Crystal structure: contains datablocks I, global. DOI: 10.1107/S1600536808018631/bx2150sup1.cif

Structure factors: contains datablocks I. DOI: 10.1107/S1600536808018631/bx2150Isup2.hkl

Additional supplementary materials:  crystallographic information; 3D view; checkCIF report

## Figures and Tables

**Table 1:** Hydrogen-bond geometry (Å, °)

*D*—H⋯*A*	*D*—H	H⋯*A*	*D*⋯*A*	*D*—H⋯*A*
O2—H10⋯Cl1^i^	0.80	2.39	3.1581 (16)	161

Symmetry code: (i) 
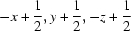
.

**Fig. 1.**
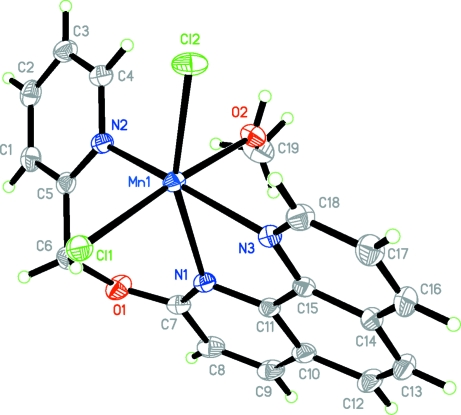
Structure of the title complex with the atom-numbering scheme. Displacement ellipsoids are drawn at the 30% probability level.

**Fig. 2.**
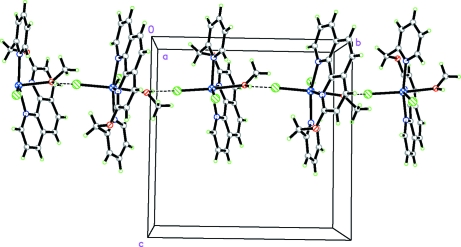
A view of the packing in the crystal structure. Intermolecular Hydrogen bonds as dashed lines.

## supplementary crystallographic information

### Comment

Derivatives of 1,10-phenanthroline play an important role in modern coordination chemistry and many complexes have been reported with different subsitituent groups (Li *et al.* 2008; Liu *et al.* 2008), but no complex with (pyridyl-2-yl)methoxy as substituent has been published. We report here the crystal structure of  the title complex, Fig1.

Compound (I) is a monomer, in which the Mn atom adopts a distorted octahedral geometry completed by two N-atom donors from 1,10-phenanthroline, one N atom from pyridine ring, two Cl atom and one O atom from methanol molecule. In neighboring monomers, there is a strong π-π interaction between 1,10-phenanthroline ligand and pyridine ring with a centroid-to-centroid distance of 3.5518 (13) Å . In addition, the crystal structure contains O—H···Cl hydrogen bonds that made the neighboring complexes connect into a one-dimensional chain along *b* axis as shown in Fig. 2.

### Experimental

10 ml methanol solution of (2-((pyridin-2-yl)methoxy)-1,10-phenanthroline (0.1200 g, 0.418 mmol) was added into 15 ml methanol solution of MnCl_2_.4H_2_O (0.0827 g, 0.418 mmol) and the mixture was stirred for a few minutes. The colorless single crystals were obtained after the filtrate had been allowed to stand at room temperature for a week.

### Refinement

H atoms from the hydroxyl group of methanol was located in a difference Fourier map with O—H = 0.80 Å and refined as riding with *U*_iso_(H) = 1.5*U*_eq_(O); Other H atoms were placed in calculated positions with C—H = 0.96 Å for methyl group, C—H = 0.97 Å for methylene group and C—H = 0.93 Å for other H atoms, and refined as riding with *U*_iso_(H) = 1.5*U*_eq_(C) for methyl group and *U*_iso_(H) = 1.2*U*_eq_(C) for other H atoms.

### Crystal data

**Table:**

[MnCl_2_(C_18_H_13_N_3_O)(CH_4_O)]	*F*_000_ = 908
*M**_r_* = 445.20	*D*_x_ = 1.589 Mg m^−^^3^
Monoclinic, *P*2_1_/*n*	Mo *K*α radiation λ = 0.71073 Å
Hall symbol: -P 2yn	Cell parameters from 3203 reflections
*a* = 10.0390 (16) Å	θ = 2.5–25.7º
*b* = 13.667 (2) Å	µ = 1.02 mm^−^^1^
*c* = 13.583 (2) Å	*T* = 298 (2) K
β = 92.874 (2)º	Block, colorless
*V* = 1861.2 (5) Å^3^	0.38 × 0.18 × 0.13 mm
*Z* = 4	

### Data collection

**Table:**

Bruker SMART APEX CCD diffractometer	4048 independent reflections
Radiation source: fine-focus sealed tube	3278 reflections with *I* > 2σ(*I*)
Monochromator: graphite	*R*_int_ = 0.033
*T* = 298(2) K	θ_max_ = 27.0º
φ and ω scans	θ_min_ = 2.1º
Absorption correction: multi-scan(SADABS; Sheldrick, 2008)	*h* = −12→12
*T*_min_ = 0.699, *T*_max_ = 0.879	*k* = −13→17
10717 measured reflections	*l* = −15→17

### Refinement

**Table:**

Refinement on *F*^2^	Secondary atom site location: difference Fourier map
Least-squares matrix: full	Hydrogen site location: inferred from neighbouring sites
*R*[*F*^2^ > 2σ(*F*^2^)] = 0.036	H-atom parameters constrained
*wR*(*F*^2^) = 0.090	*w* = 1/[σ^2^(*F*_o_^2^) + (0.0466*P*)^2^] where *P* = (*F*_o_^2^ + 2*F*_c_^2^)/3
*S* = 1.01	(Δ/σ)_max_ = 0.001
4048 reflections	Δρ_max_ = 0.29 e Å^−^^3^
245 parameters	Δρ_min_ = −0.25 e Å^−^^3^
1 restraint	Extinction correction: none
Primary atom site location: structure-invariant direct methods	

### Special details

**Table:**

Geometry. All e.s.d.'s (except the e.s.d. in the dihedral angle between two l.s. planes) are estimated using the full covariance matrix. The cell e.s.d.'s are taken into account individually in the estimation of e.s.d.'s in distances, angles and torsion angles; correlations between e.s.d.'s in cell parameters are only used when they are defined by crystal symmetry. An approximate (isotropic) treatment of cell e.s.d.'s is used for estimating e.s.d.'s involving l.s. planes.
Refinement. Refinement of *F*^2^ against ALL reflections. The weighted *R*-factor *wR* and goodness of fit *S* are based on *F*^2^, conventional *R*-factors *R* are based on *F*, with *F* set to zero for negative *F*^2^. The threshold expression of *F*^2^ > σ(*F*^2^) is used only for calculating *R*-factors(gt) *etc*. and is not relevant to the choice of reflections for refinement. *R*-factors based on *F*^2^ are statistically about twice as large as those based on *F*, and *R*- factors based on ALL data will be even larger.

### Fractional atomic coordinates and isotropic or equivalent isotropic displacement parameters (Å^2^)

**Table:**

	*x*	*y*	*z*	*U*_iso_*/*U*_eq_	
C1	0.1526 (2)	0.79472 (15)	0.58696 (17)	0.0372 (5)	
H1	0.0945	0.7746	0.6342	0.045*	
C2	0.2736 (2)	0.83666 (16)	0.61481 (17)	0.0409 (6)	
H2	0.2988	0.8449	0.6811	0.049*	
C3	0.3567 (2)	0.86618 (16)	0.54275 (17)	0.0399 (5)	
H3	0.4390	0.8944	0.5596	0.048*	
C4	0.3163 (2)	0.85338 (16)	0.44621 (17)	0.0373 (5)	
H4	0.3726	0.8743	0.3981	0.045*	
C5	0.1191 (2)	0.78311 (14)	0.48767 (16)	0.0308 (5)	
C6	−0.01179 (19)	0.73705 (16)	0.45422 (16)	0.0355 (5)	
H6A	−0.0034	0.7067	0.3903	0.043*	
H6B	−0.0349	0.6865	0.5005	0.043*	
C7	−0.1580 (2)	0.84967 (15)	0.36073 (17)	0.0331 (5)	
C8	−0.2826 (2)	0.89726 (17)	0.36516 (18)	0.0409 (6)	
H8	−0.3257	0.9010	0.4241	0.049*	
C9	−0.3375 (2)	0.93711 (16)	0.28180 (19)	0.0420 (6)	
H9	−0.4195	0.9687	0.2829	0.050*	
C10	−0.2712 (2)	0.93111 (14)	0.19319 (18)	0.0361 (5)	
C11	−0.14608 (19)	0.88455 (14)	0.19628 (16)	0.0293 (5)	
C12	−0.3287 (2)	0.96695 (16)	0.1018 (2)	0.0461 (6)	
H12	−0.4100	0.9996	0.1010	0.055*	
C13	−0.2673 (2)	0.95437 (17)	0.0167 (2)	0.0476 (6)	
H13	−0.3079	0.9764	−0.0422	0.057*	
C14	−0.1406 (2)	0.90753 (15)	0.01644 (17)	0.0375 (5)	
C15	−0.0780 (2)	0.87583 (14)	0.10597 (16)	0.0309 (5)	
C16	−0.0753 (3)	0.88884 (18)	−0.07041 (18)	0.0499 (7)	
H16	−0.1147	0.9070	−0.1311	0.060*	
C17	0.0457 (3)	0.84415 (18)	−0.06574 (18)	0.0474 (6)	
H17	0.0891	0.8303	−0.1230	0.057*	
C18	0.1037 (2)	0.81939 (16)	0.02599 (17)	0.0395 (5)	
H18	0.1878	0.7907	0.0286	0.047*	
C19	0.0884 (3)	1.03698 (18)	0.3451 (2)	0.0593 (7)	
H19A	−0.0056	1.0389	0.3283	0.089*	
H19B	0.1245	1.1019	0.3419	0.089*	
H19C	0.1031	1.0117	0.4106	0.089*	
Cl1	0.10996 (5)	0.62442 (4)	0.24263 (4)	0.03925 (15)	
Cl2	0.36834 (5)	0.82286 (5)	0.19848 (5)	0.04570 (17)	
Mn1	0.14453 (3)	0.80494 (2)	0.25537 (2)	0.02795 (11)	
N1	−0.08860 (16)	0.84415 (12)	0.28120 (13)	0.0288 (4)	
N2	0.19883 (16)	0.81198 (12)	0.41697 (13)	0.0314 (4)	
N3	0.04509 (16)	0.83459 (12)	0.10973 (13)	0.0304 (4)	
O1	−0.11631 (14)	0.81011 (12)	0.44771 (12)	0.0424 (4)	
O2	0.15254 (14)	0.97535 (10)	0.27729 (11)	0.0372 (4)	
H10	0.2227	1.0012	0.2676	0.056*	

### Atomic displacement parameters (Å^2^)

**Table:**

	*U*^11^	*U*^22^	*U*^33^	*U*^12^	*U*^13^	*U*^23^
C1	0.0430 (13)	0.0372 (13)	0.0317 (12)	0.0068 (10)	0.0055 (10)	0.0042 (9)
C2	0.0529 (15)	0.0374 (13)	0.0315 (13)	0.0060 (11)	−0.0074 (11)	−0.0010 (10)
C3	0.0361 (12)	0.0417 (14)	0.0408 (14)	−0.0015 (10)	−0.0076 (10)	−0.0019 (10)
C4	0.0295 (11)	0.0426 (13)	0.0397 (14)	−0.0022 (9)	−0.0004 (10)	0.0040 (10)
C5	0.0294 (11)	0.0290 (11)	0.0340 (12)	0.0056 (8)	0.0021 (9)	0.0025 (9)
C6	0.0322 (11)	0.0375 (12)	0.0372 (13)	0.0016 (9)	0.0063 (9)	0.0059 (10)
C7	0.0260 (10)	0.0348 (12)	0.0386 (13)	−0.0033 (9)	0.0006 (9)	−0.0047 (9)
C8	0.0259 (11)	0.0466 (14)	0.0508 (15)	0.0023 (10)	0.0083 (10)	−0.0090 (11)
C9	0.0253 (11)	0.0357 (13)	0.0647 (17)	0.0054 (9)	−0.0015 (11)	−0.0082 (11)
C10	0.0283 (11)	0.0253 (11)	0.0539 (15)	0.0003 (9)	−0.0058 (10)	−0.0022 (10)
C11	0.0261 (10)	0.0225 (10)	0.0389 (13)	−0.0022 (8)	−0.0037 (9)	−0.0016 (8)
C12	0.0345 (12)	0.0364 (13)	0.0657 (18)	0.0071 (10)	−0.0153 (12)	0.0040 (12)
C13	0.0455 (14)	0.0414 (14)	0.0538 (17)	0.0008 (11)	−0.0184 (12)	0.0109 (11)
C14	0.0389 (12)	0.0306 (11)	0.0420 (14)	−0.0058 (10)	−0.0092 (10)	0.0066 (10)
C15	0.0290 (11)	0.0253 (11)	0.0376 (13)	−0.0056 (8)	−0.0043 (9)	0.0005 (9)
C16	0.0564 (16)	0.0565 (16)	0.0355 (15)	−0.0093 (13)	−0.0107 (12)	0.0141 (11)
C17	0.0545 (16)	0.0569 (16)	0.0309 (13)	−0.0074 (13)	0.0024 (11)	0.0023 (11)
C18	0.0364 (12)	0.0465 (14)	0.0356 (13)	−0.0016 (10)	0.0030 (10)	−0.0020 (10)
C19	0.0622 (17)	0.0401 (15)	0.078 (2)	−0.0018 (12)	0.0270 (15)	−0.0142 (13)
Cl1	0.0355 (3)	0.0310 (3)	0.0508 (4)	0.0014 (2)	−0.0029 (2)	−0.0018 (2)
Cl2	0.0283 (3)	0.0637 (4)	0.0460 (4)	−0.0058 (3)	0.0106 (2)	−0.0148 (3)
Mn1	0.02338 (17)	0.03248 (19)	0.02796 (19)	0.00096 (12)	0.00103 (13)	−0.00058 (13)
N1	0.0243 (9)	0.0298 (9)	0.0322 (10)	0.0001 (7)	−0.0003 (7)	−0.0021 (7)
N2	0.0266 (9)	0.0361 (10)	0.0313 (10)	−0.0010 (7)	0.0000 (8)	0.0022 (7)
N3	0.0273 (9)	0.0324 (10)	0.0312 (10)	−0.0013 (7)	−0.0004 (7)	0.0011 (7)
O1	0.0303 (8)	0.0628 (11)	0.0345 (9)	0.0104 (7)	0.0074 (7)	0.0018 (7)
O2	0.0357 (8)	0.0323 (8)	0.0443 (9)	−0.0042 (6)	0.0076 (7)	−0.0018 (7)

### Geometric parameters (Å, °)

**Table:**

C1—C2	1.379 (3)	C12—C13	1.347 (3)
C1—C5	1.382 (3)	C12—H12	0.9300
C1—H1	0.9300	C13—C14	1.424 (3)
C2—C3	1.377 (3)	C13—H13	0.9300
C2—H2	0.9300	C14—C16	1.402 (3)
C3—C4	1.364 (3)	C14—C15	1.409 (3)
C3—H3	0.9300	C15—N3	1.357 (3)
C4—N2	1.350 (3)	C16—C17	1.358 (3)
C4—H4	0.9300	C16—H16	0.9300
C5—N2	1.340 (3)	C17—C18	1.390 (3)
C5—C6	1.507 (3)	C17—H17	0.9300
C6—O1	1.448 (2)	C18—N3	1.323 (3)
C6—H6A	0.9700	C18—H18	0.9300
C6—H6B	0.9700	C19—O2	1.425 (3)
C7—N1	1.316 (3)	C19—H19A	0.9600
C7—O1	1.347 (3)	C19—H19B	0.9600
C7—C8	1.414 (3)	C19—H19C	0.9600
C8—C9	1.349 (3)	Cl1—Mn1	2.4961 (7)
C8—H8	0.9300	Cl2—Mn1	2.4248 (7)
C9—C10	1.407 (3)	Mn1—N3	2.2082 (18)
C9—H9	0.9300	Mn1—N2	2.2370 (18)
C10—C11	1.407 (3)	Mn1—O2	2.3487 (14)
C10—C12	1.430 (3)	Mn1—N1	2.4434 (17)
C11—N1	1.379 (3)	O2—H10	0.8048
C11—C15	1.439 (3)		
			
C2—C1—C5	118.9 (2)	N3—C15—C11	118.23 (18)
C2—C1—H1	120.6	C14—C15—C11	119.99 (19)
C5—C1—H1	120.6	C17—C16—C14	119.8 (2)
C3—C2—C1	118.9 (2)	C17—C16—H16	120.1
C3—C2—H2	120.6	C14—C16—H16	120.1
C1—C2—H2	120.6	C16—C17—C18	119.0 (2)
C4—C3—C2	119.0 (2)	C16—C17—H17	120.5
C4—C3—H3	120.5	C18—C17—H17	120.5
C2—C3—H3	120.5	N3—C18—C17	123.2 (2)
N2—C4—C3	123.3 (2)	N3—C18—H18	118.4
N2—C4—H4	118.3	C17—C18—H18	118.4
C3—C4—H4	118.3	O2—C19—H19A	109.5
N2—C5—C1	122.74 (19)	O2—C19—H19B	109.5
N2—C5—C6	116.75 (19)	H19A—C19—H19B	109.5
C1—C5—C6	120.5 (2)	O2—C19—H19C	109.5
O1—C6—C5	110.41 (17)	H19A—C19—H19C	109.5
O1—C6—H6A	109.6	H19B—C19—H19C	109.5
C5—C6—H6A	109.6	N3—Mn1—N2	161.46 (6)
O1—C6—H6B	109.6	N3—Mn1—O2	86.71 (6)
C5—C6—H6B	109.6	N2—Mn1—O2	80.09 (6)
H6A—C6—H6B	108.1	N3—Mn1—Cl2	94.62 (5)
N1—C7—O1	122.89 (18)	N2—Mn1—Cl2	97.14 (5)
N1—C7—C8	124.5 (2)	O2—Mn1—Cl2	85.06 (4)
O1—C7—C8	112.6 (2)	N3—Mn1—N1	72.25 (6)
C9—C8—C7	118.4 (2)	N2—Mn1—N1	92.16 (6)
C9—C8—H8	120.8	O2—Mn1—N1	77.95 (5)
C7—C8—H8	120.8	Cl2—Mn1—N1	158.93 (5)
C8—C9—C10	120.2 (2)	N3—Mn1—Cl1	93.68 (5)
C8—C9—H9	119.9	N2—Mn1—Cl1	97.86 (5)
C10—C9—H9	119.9	O2—Mn1—Cl1	173.00 (4)
C11—C10—C9	117.5 (2)	Cl2—Mn1—Cl1	101.86 (2)
C11—C10—C12	120.1 (2)	N1—Mn1—Cl1	95.50 (4)
C9—C10—C12	122.4 (2)	C7—N1—C11	116.55 (17)
N1—C11—C10	122.8 (2)	C7—N1—Mn1	132.87 (14)
N1—C11—C15	118.89 (17)	C11—N1—Mn1	109.35 (13)
C10—C11—C15	118.28 (19)	C5—N2—C4	117.19 (19)
C13—C12—C10	121.3 (2)	C5—N2—Mn1	124.45 (13)
C13—C12—H12	119.4	C4—N2—Mn1	118.23 (14)
C10—C12—H12	119.4	C18—N3—C15	118.45 (19)
C12—C13—C14	120.5 (2)	C18—N3—Mn1	122.74 (14)
C12—C13—H13	119.8	C15—N3—Mn1	118.68 (14)
C14—C13—H13	119.8	C7—O1—C6	121.52 (17)
C16—C14—C15	117.6 (2)	C19—O2—Mn1	130.86 (14)
C16—C14—C13	122.7 (2)	C19—O2—H10	105.9
C15—C14—C13	119.7 (2)	Mn1—O2—H10	116.0
N3—C15—C14	121.8 (2)		
			
C5—C1—C2—C3	0.5 (3)	Cl1—Mn1—N1—C7	87.71 (18)
C1—C2—C3—C4	0.3 (3)	N3—Mn1—N1—C11	−13.64 (12)
C2—C3—C4—N2	−0.8 (3)	N2—Mn1—N1—C11	156.13 (12)
C2—C1—C5—N2	−0.8 (3)	O2—Mn1—N1—C11	76.75 (12)
C2—C1—C5—C6	179.61 (19)	Cl2—Mn1—N1—C11	39.8 (2)
N2—C5—C6—O1	−93.6 (2)	Cl1—Mn1—N1—C11	−105.76 (12)
C1—C5—C6—O1	86.0 (2)	C1—C5—N2—C4	0.4 (3)
N1—C7—C8—C9	2.4 (3)	C6—C5—N2—C4	179.96 (18)
O1—C7—C8—C9	−177.8 (2)	C1—C5—N2—Mn1	−175.49 (14)
C7—C8—C9—C10	−0.1 (3)	C6—C5—N2—Mn1	4.1 (2)
C8—C9—C10—C11	−1.4 (3)	C3—C4—N2—C5	0.4 (3)
C8—C9—C10—C12	176.2 (2)	C3—C4—N2—Mn1	176.55 (17)
C9—C10—C11—N1	1.0 (3)	N3—Mn1—N2—C5	67.7 (3)
C12—C10—C11—N1	−176.65 (18)	O2—Mn1—N2—C5	112.92 (16)
C9—C10—C11—C15	178.68 (18)	Cl2—Mn1—N2—C5	−163.39 (15)
C12—C10—C11—C15	1.0 (3)	N1—Mn1—N2—C5	35.55 (16)
C11—C10—C12—C13	2.2 (3)	Cl1—Mn1—N2—C5	−60.30 (16)
C9—C10—C12—C13	−175.4 (2)	N3—Mn1—N2—C4	−108.1 (2)
C10—C12—C13—C14	−2.1 (3)	O2—Mn1—N2—C4	−62.89 (15)
C12—C13—C14—C16	177.1 (2)	Cl2—Mn1—N2—C4	20.79 (15)
C12—C13—C14—C15	−1.2 (3)	N1—Mn1—N2—C4	−140.26 (15)
C16—C14—C15—N3	4.4 (3)	Cl1—Mn1—N2—C4	123.88 (15)
C13—C14—C15—N3	−177.20 (19)	C17—C18—N3—C15	0.4 (3)
C16—C14—C15—C11	−174.03 (19)	C17—C18—N3—Mn1	−175.35 (17)
C13—C14—C15—C11	4.4 (3)	C14—C15—N3—C18	−3.7 (3)
N1—C11—C15—N3	−4.9 (3)	C11—C15—N3—C18	174.77 (18)
C10—C11—C15—N3	177.29 (17)	C14—C15—N3—Mn1	172.26 (14)
N1—C11—C15—C14	173.53 (17)	C11—C15—N3—Mn1	−9.3 (2)
C10—C11—C15—C14	−4.2 (3)	N2—Mn1—N3—C18	154.10 (19)
C15—C14—C16—C17	−1.8 (3)	O2—Mn1—N3—C18	109.61 (17)
C13—C14—C16—C17	179.8 (2)	Cl2—Mn1—N3—C18	24.84 (16)
C14—C16—C17—C18	−1.2 (4)	N1—Mn1—N3—C18	−172.00 (18)
C16—C17—C18—N3	2.0 (4)	Cl1—Mn1—N3—C18	−77.39 (16)
O1—C7—N1—C11	177.43 (18)	N2—Mn1—N3—C15	−21.6 (3)
C8—C7—N1—C11	−2.8 (3)	O2—Mn1—N3—C15	−66.13 (14)
O1—C7—N1—Mn1	−16.8 (3)	Cl2—Mn1—N3—C15	−150.90 (14)
C8—C7—N1—Mn1	162.96 (16)	N1—Mn1—N3—C15	12.26 (13)
C10—C11—N1—C7	1.1 (3)	Cl1—Mn1—N3—C15	106.87 (14)
C15—C11—N1—C7	−176.61 (17)	N1—C7—O1—C6	−16.9 (3)
C10—C11—N1—Mn1	−167.94 (15)	C8—C7—O1—C6	163.28 (18)
C15—C11—N1—Mn1	14.4 (2)	C5—C6—O1—C7	100.3 (2)
N3—Mn1—N1—C7	179.82 (19)	N3—Mn1—O2—C19	114.2 (2)
N2—Mn1—N1—C7	−10.41 (18)	N2—Mn1—O2—C19	−52.8 (2)
O2—Mn1—N1—C7	−89.79 (18)	Cl2—Mn1—O2—C19	−150.9 (2)
Cl2—Mn1—N1—C7	−126.75 (17)	N1—Mn1—O2—C19	41.6 (2)

### Hydrogen-bond geometry (Å, °)

**Table:**

*D*—H···*A*	*D*—H	H···*A*	*D*···*A*	*D*—H···*A*
O2—H10···Cl1^i^	0.80	2.39	3.1581 (16)	161

Symmetry codes: (i) −*x*+1/2, *y*+1/2, −*z*+1/2.
